# Efficacy and safety of pembrolizumab versus sintilimab treatment in patients with advanced squamous lung cancer: A real-world study in China

**DOI:** 10.3389/fonc.2023.1147903

**Published:** 2023-04-12

**Authors:** Wenyu Yang, Tao Li, Yibing Bai, Yaping Long, Ming Gao, Ting Wang, Fangfang Jing, Fan Zhang, Haitao Tao, Junxun Ma, Lijie Wang, Yi Hu

**Affiliations:** ^1^ School of Medicine, Nankai University, Tianjin, China; ^2^ Department of Medical Oncology, Senior Department of Oncology, The Fifth Medical Center, The General Hospital of the People's Liberation Army, Beijing, China; ^3^ Department of Oncology, The First Medical Center, The General Hospital of the People's Liberation Army, Beijing, China

**Keywords:** squamous lung cancer, pembrolizumab, sintilimab, efficacy, safety

## Abstract

**Importance:**

Both pembrolizumab and sintilimab have been approved by the Chinese State Drug Administration (NMPA) for the first-line treatment of patients with advanced squamous lung cancer. The differences of the two drugs in efficacy and safety are unclear.

**Objectives:**

To compare the real-world efficacy and safety of first-line treatments in patients with advanced squamous lung cancer.

**Materials and methods:**

This was a retrospective review of patients with advanced squamous carcinoma who received sintilimab or pembrolizumab in combination with chemotherapy as first-line therapy between June 2018 and April 2022 in the Chinese PLA Hospital. The primary objective was to compare the objective response rate (ORR), progression-free survival (PFS), and overall survival (OS) between the two groups. Secondary objectives were to compare the disease control rate (DCR) and to analyze adverse events (AEs) between the two groups.

**Results:**

A total of 164 patients were enrolled, including 63 patients (38.4%) in the sintilimab-combined chemotherapy group and 101 patients (61.6%) in the pembrolizumab-combined chemotherapy group. The ORR was 65.10% in the sintilimab group and 61.40% in the pembrolizumab group (P=0.634). The DCR was 92.10% and 92.10% in the sintilimab and pembrolizumab groups, respectively (P=0.991). The median PFS was 22.2 months for patients treated with sintilimab group compared with 16.5 months for patients treated with pembrolizumab group[hazard ratio (HR) = 0.743; 95% confidence interval (CI): 0.479-1.152; P = 0.599]. Patients treated with pembrolizumab did not achieve a median OS, and patients treated with sintilimab had a median OS of 30.7 months. In the sintilimab group, the incidence of all treatment-related adverse events (TRAEs) was 92.1% (58/63), and the incidence of grade 3-4 TRAEs of 42.9% (27/63). In the pembrolizumab group, the incidence of all TRAEs was 90.1% (91/101), and the incidence of grade 3-4 TRAEs was 37.6% (38/101).

**Conclusion:**

In the clinical treatment of Chinese patients with advanced squamous lung cancer, first-line treatment with sintilimab in combination with chemotherapy provided similar efficacy to pembrolizumab in combination with chemotherapy, and the treatment-related adverse effect profiles were comparable between the two groups, including similar rates of grade 3-4 and all adverse events.

## 1 Introduction

Lung cancer is a malignant neoplastic disease with the highest mortality rate in the world today (1). Non-small cell lung cancer (NSCLC), the most common histologic type, accounts for more than 85% of all lung cancers. Squamous lung cancer cases account for approximately 17% of all NSCLC cases (2). Advanced squamous lung cancer patients have poor prognosis, who receive treatment with platinum-based regimens struggling to achieve the one-year overall survival time. Programmed cell death protein 1 (PD1) is one of the checkpoints that regulates the immune response. Currently, immune checkpoint inhibitors (ICIs), such as programmed death 1 (PD-1) and programmed death ligand 1 (PD-L1) inhibitors, have been widely used in clinical practice, showing good efficacy and safety in a variety of tumors (3). ICIs also bring a new opportunity for the treatment of patients with advanced squamous lung cancer. Studies have shown that immunotherapy in combination with chemotherapy can result in significant improvements in patients, which may be related to the immunological effects mediated by chemotherapeutic agents through direct and indirect stimulation of immune responses and increased tumor immunogenicity.

Pembrolizumab is a humanized monoclonal anti-PD-1 antibody that has been widely used in the clinical treatment of a variety of malignancies. The KEYNOTE-407 clinical trial demonstrated that pembrolizumab in combination with platinum-based therapies can be the standard of treatment in the first-line treatment of advanced squamous lung cancer, regardless of PD-L1 expression (4–6). Sintilimab is a fully human IgG4 monoclonal antibody, which has a unique PD-1 epitope that blocks the binding of PD-1 to PD-L1 and PD-L2 (7). Based on the ORIENT-12 clinical trial, sintilimab in combination with gemcitabine and platinum-based therapies was approved by the NMPA for the first-line treatment of nonsurgically resectable locally advanced or metastatic squamous lung cancer (8). The NMPA has approved platinum-based therapies for the first-line treatment of inoperable advanced or metastatic squamous lung cancer. According to the latest Chinese Society of Clinical Oncology (CSCO) 2022 guidelines on clinical practice of immune checkpoint inhibitors and CSCO guidelines on NSCLC, both pembrolizumab in combination with chemotherapy and sintilimab in combination with platinum-based chemotherapy are recommended as Class 1A first-line therapy for patients with advanced squamous lung cancer without driver mutations.

In KEYNOTE-407 and ORIENT-12 clinical trials, both pembrolizumab and sintilimab showed good efficacy and safety in the treatment of advanced squamous lung cancer, but the clinical trial populations were different, with pembrolizumab being used in a predominantly non-Asian population and sintilimab in a predominantly Chinese population. The binding sites and biological activities of pembrolizumab and sintilimab are different. There is a lack of real-world comparative studies on the efficacy and safety of different immunotherapeutic agents in patients with advanced squamous lung cancer. Therefore, we conducted a retrospective cohort study to compare the efficacy and safety of real-world treatment with sintilimab and pembrolizumab as first-line therapy in patients with advanced squamous lung cancer.

## 2 Materials and methods

### 2.1 Patient characteristics

This retrospective study was conducted in patients with advanced squamous lung cancer who received consecutive chemotherapy in combination with sintilimab or pembrolizumab as first-line treatment at the Chinese PLA general hospital (Beijing, China) between June 2018 and April 2022. The inclusion criteria were as follows: 1) pathologically definite diagnosis of squamous epithelial cell carcinoma of the lung; 2) patients with advanced squamous non-small cell lung cancer of stage IIIB-IV according to the International Association for the Study of Lung Cancer (IASLC) TNM Staging of Lung Cancer (8th edition) and relevant imaging; and 3) patients who received at least 2 cycles of sintilimab or pembrolizumab in combination with chemotherapy in first-line treatment; 4) patients with lesions available for imaging measurements and evaluation for efficacy; and 5) ECOG score ≤ 2. Exclusion criteria were as follows: 1) lack of clear pathological diagnosis; 2) lung squamous carcinoma combined with other malignancies; and 3) patients who have received previous antineoplastic therapy. As this study was retrospective, a waiver of personal consent was allowed. All procedures performed in this study were in accordance with the Declaration of Helsinki (revised 2013).

### 2.2 Treatment options

Patients were treated with either pembrolizumab (200 mg every 3 weeks over 30 min IV infusion) or sintilimab (200 mg every 3 weeks IV infusion). The chemotherapeutic drug regimens were platinum-based dual drug regimen, including gemcitabine in combination with cisplatin or carboplatin and paclitaxel in combination with cisplatin or carboplatin, chosen by the clinician on a case-by-case basis. Chemotherapeutic agents were administered as follows: gemcitabine 1,250 mg/m^2^, intravenously; albumin-bound paclitaxel 260 mg/m^2^, intravenously; paclitaxel 175 mg/m^2^, intravenously; cisplatin 75 mg/m^2^, intravenously; carboplatin AUC 5 mg/ml/min, intravenously.

### 2.3 Assessment

Basic patient characteristics and clinical information were collected, including age, sex, smoking history, Eastern Cooperative Oncology Group physical status (ECOG-PS), tumor TNM stage, histologic type, metastases, PD-L1 expression, number of treatment cycles, time to progression, time to death, and adverse events. Computed tomography (CT) scans of the chest and abdomen and magnetic resonance imaging (MRI) of the head were collected and evaluated for efficacy according to the Response Evaluation Criteria in Solid Tumors (RECIST, version 1.1) definition. Efficacy evaluation included complete response (CR), partial response (PR), stable disease (SD), and progressive disease (PD). The objective response rate (ORR) was defined as (CR+PR)/(CR+PR+SD+PD)×100%; the disease control rate (DCR) was defined as (CR+PR+SD)/(CR+PR+SD+PD)×100%. Progression-free survival (PFS) was defined as the time interval between the start of first-line treatment and disease progression or death; overall survival (OS) was defined as the time between the start of first-line treatment and death from any cause. PFS data or OS data were censored for patients who had not progressed, were lost to follow-up or were still alive at the end of the follow-up period. The follow-up cutoff date was August 24, 2022. Evaluation of all adverse events: Adverse reactions were evaluated according to the Common Terminology Criteria for Adverse Events (CTCAE) version 4.0 (Class I-IV).

### 2.4 Statistical analysis

SPSS 26.0 was used for statistical analysis. Categorical variables were expressed as frequencies and percentages, and continuous variables were expressed as medians and ranges. Baseline characteristics and efficacy data of the two treatment groups were compared using the χ2 test or Wilcoxon rank sum test. Kaplan-Meier survival models were developed, and PFS and OS were compared between the two groups using the log-rank test. For subgroup analysis, PFS and OS were calculated using the same method after classifying patients by age, sex, smoking status, ECOG-PS, tumor TNM stage, pathological type, PD-L1 expression, and treatment strategy. Differences with p values < 0.05 were considered statistically significant differences.

## 3 Results

### 3.1 Patient baseline information characteristics

A total of 164 patients with advanced squamous lung cancer receiving pembrolizumab or sintilimab in combination with chemotherapy as first-line therapy were enrolled in this study. **Table 1** shows the baseline characteristics of the patients. Sixty-three patients (38.4%) were in the sintilimab group, and 101 patients (61.6%) were in the pembrolizumab monotherapy group. The baseline characteristics of the patients in both groups were comparable.

**Table 1:** Baseline characteristics of the study participants.

Characteristic	Pembrolizumab (N=101)	Sintilimab (N=63)	P value
**Median age (range), years**	65 (55-74)	65 (57-72)	
**Age, years**			0.246
≥65	54(53.5%)	27(42.9%)	
<65	47(46.5%)	36(57.1%)	
**Sex**			0.484
Male	94 (93.1%)	61 (96.8%)	
Female	7 (6.9%)	2 (3.2%)	
**Smoking history**			0.089
Never	29 (28.7%)	9 (14.3%)	
Current	5 (5.0%)	3 (4.8%)	
Past	67 (66.3%)	51 (81.0%)	
**Stage**			0.744
IIIB/IIIC	16 (15.8%)	8 (12.7%)	
IV	85 (84.2%)	55 (87.3%)	
**Family History**			0.563
Yes	25 (24.7%)	19 (30.2%)	
No	76 (73.3%)	44 (69.8%)	
**Metastasis**			0.311
Brain	10 (9.9%)	3 (4.8%)	
Bone	16 (15.8%)	16 (25.4%)	
Liver	7 (6.9%)	5 (7.9%)	
Adrenal gland	9 (8.9%)	3 (4.8%)	
Pleural	6 (5.9%)	6 (9.5%)	
**PD-L1 expression**			0.455
Not examined	48 (47.5%)	37 (58.7%)	
<1%	17 (16.8%)	7 (11.1%)	
≥1%	36(35.6%)	19(30.1%)	
1%-49%	26 (25.7%)	12 (19.0%)	
≥50%	10 (9.9%)	7 (11.1%)	
**Combination of chemotherapy**			
Gemcitabine+Cisplatin	5(5.0%)	3(5.0%)	
Gemcitabine+Carboplatin	2(2.0%)	0	
Paclitaxel+Cisplatin	74(73.3%)	42(66.7%)	
Paclitaxel+Carboplatin	20(19.8%)	18(28.6%)	
**Combination of radiotherapy**			0.867
Yes	20 (19.8%)	11 (17.5%)	
No	81 (80.2%)	52 (82.5%)	

The median age was 65 years (57-72 years) in the sintilimab group and 65 years (55-74 years) in the pembrolizumab group. The proportion of men was higher than that of women in both groups. 54 patients in the sintilimab group and 72 patients in the pembrolizumab group were past or current smokers. Stage IV patients were predominant in both groups, with 55 (87.3%) and 85 (84.2%) patients; there were 8 (12.7%) and 16 (15.8%) and IIIB/IIIC patients in the sintilimab and pembrolizumab groups, respectively. A total of 44 patients with squamous lung cancer had a family history, including 19 in the sintilimab group and 25 in the pembrolizumab group. There were 48 patients with distant metastases in the sintilimab group; 16 patients (25.4%) had bone metastases, 3 (4.8%) had brain metastases, 5 (7.9%) had liver metastases, 3 (4.8%) had adrenal metastases, and 6 (9.5%) had pleural metastases. There were 86 patients with distant metastases in the pembrolizumab group; 16 (15.8%) had bone metastases, 10 (9.9%) had brain metastases, 7 (6.9%) had liver metastases, 9 (8.9%) had adrenal metastases, and 6 (5.9%) had pleural metastases. A total of 79 patients underwent a PD-L1 (22C3) expression assay before treatment. In the sintilimab group, 7 patients had high PD-L1 expression (PD-L1 ≥ 50%), 12 patients had low PD-L1 expression (1% ≤ PD-L1 < 50%), and 7 patients had negative PD-L1 expression (PD-L1 < 1%). In the pembrolizumab group, 10 patients had high PD-L1 expression (PD-L1 ≥ 50%), 26 had low PD-L1 expression (1% ≤ PD-L1 < 50%), and 17 had negative PD-L1 expression (PD-L1 < 1%) (**Table 1**).

### 3.2 Recent results

In the sintilimab group, 41 (65.1%) patients achieved PR, 17 (27.0%) patients achieved SD, and 5 (7.9%) patients developed PD. In the pembrolizumab group, 62 (61.3%) patients developed PR, 31 (30.7%) patients developed SD, and 8 (7.9%) patients developed PD. The ORRs in the sintilimab and pembrolizumab groups were 65.1% and 61.4% (P=0.634), and the DCRs were 92.0% and 92.0% (P=0.991), respectively (**Table 2**).

**Table 2:** Comparison of short-term clinical outcomes between the two groups.

Best overall response	Pembrolizumab (N=101)	Sintilimab (N=63)	P value
CR	0	0	
PR	62	41	
SD	31	17	
PD	8	5	
ORR%	61.40%	65.10%	0.634
DCR%	92.10%	92.10%	0.991

### 3.3 Long-term survival

**Figure 1:**
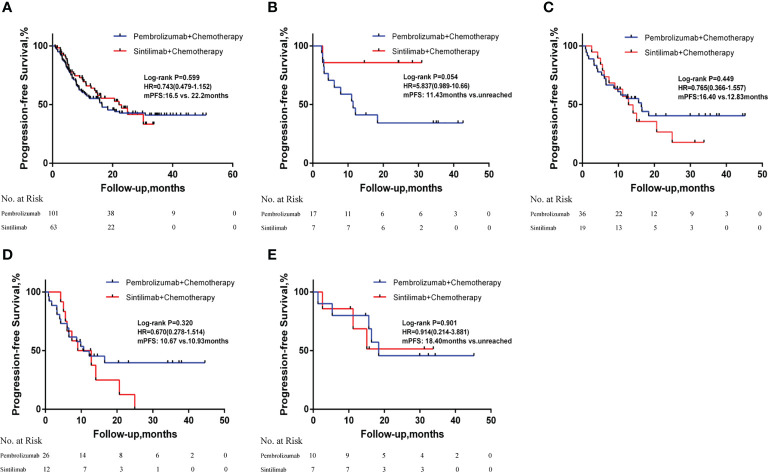
Kaplan-Meier curves of progression-free survival in **(A)** all patients; **(B)**patients with negative PD-L1 expression; **(C)** patients with positive PD-L1 expression; **(D)** patients with low PD-L1 expression and **(E)** patients with high PD-L1 expression.HR, hazard ratios; mPFS, median progression-free survival; PD-L1, programmed death-ligand 1.

**Figure 2:**
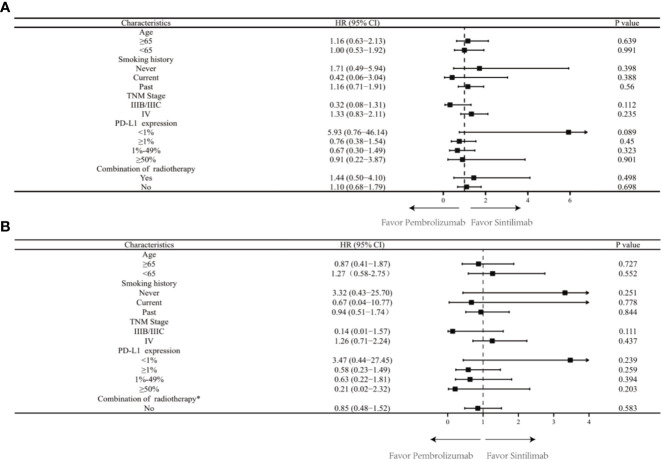
**(A)** Progression-free survival by subgroup in the full analysis set. TMN, tumor, node, metastasis; PD-L1, programmed death-ligand 1; HR, hazard ratios; CI, confidence interval; **(B)** Overall survival by subgroup in the full analysis set. TMN, tumor, node, metastasis; PD-L1, programmed death-ligand 1; HR, hazard ratios; CI, confidence interval.*Data not presented for subgroups of “Yes in Combination of radiotherapy” owing to very few patients which precludes any meaningful analysis.

There was a median PFS of 22.20 months in the sintilimab group and a median PFS of 16.50 months in the pembrolizumab group (HR = 0.734; 95% CI: 0.479-1.152; P = 0.599). In patients with negative PD-L1 expression, median PFS was not achieved after sintilimab treatment compared with a median PFS of 11.43 months after pembrolizumab treatment (HR = 5.837; 95% CI: 0.989-10.66; P=0.054). In patients with positive PD-L1 expression, the median PFS after sintilimab treatment was 12.83 months compared with a median PFS of 16.40 months with pembrolizumab treatment (HR = 0.765; 95% CI: 0.366-1.557; P=0.449). Subgroup analysis based on PD-L1 expression showed that patients with high PD-L1 expression did not achieve median PFS after treatment with sintilimab compared with a median PFS of 18.40 months for patients treated with pembrolizumab (HR = 0.914; 95% CI: 0.214-3.881; P = 0.901). In patients with low PD-L1 expression, the median PFS was 10.93 months after sintilimab treatment compared with a median PFS of 10.67 months after pembrolizumab treatment (HR = 0670; 95% CI: 0.278-1.514; P = 0.320; **Figure 1**). Subgroup analysis based on age, smoking status, tumor stage, PD-L1 expression level, and whether or not to combine chemotherapy revealed no significant difference in PFS between patients in the sintilimab and pembrolizumab groups (**Figure 2A**).

**Figure 3:**
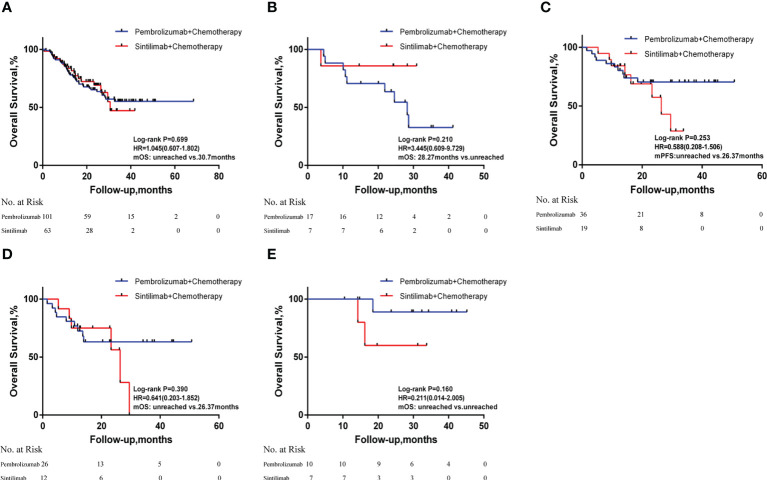
Kaplan-Meier curves of overall survival in **(A)** all patients; **(B)** patients with negative PD-L1 expression; **(C)** patients with positive PD-L1 expression; **(D)** patients with low PD-L1 expression and **(E)** patients with high PD-L1 expression. HR, hazard ratios; mOS, median overall survival; PD-L1, programmed death-ligand 1.

Overall survival analysis revealed a median OS of 30.70 months in the sintilimab group and a median OS not reached in the pembrolizumab group (HR = 1.045; 95% CI: 0.607-1.802; P=0.699). Subgroup analysis based on PD-L1 expression showed that median OS after sintilimab treatment was not achieved in patients with negative PD-L1 expression, while median OS in the pembrolizumab treatment group was 28.27 months (HR = 3.445; 95% CI: 0.609-9.729; P=0.210). In patients with positive PD-L1 expression, the median OS was not reached after pembrolizumab treatment, while the median OS in the sintilimab treatment group was 26.37 months (HR = 0.588; 95% CI: 0.208-1.506; P=0.253). Neither sintilimab nor pembrolizumab treatment-group patients achieved median OS if their levels of PD-L1 expression were high (HR = 0.211; 95% CI: 0.014-2.005; P=0.160). In patients with low PD-L1 expression, the median OS was 26.37 months after sintilimab treatment, while the median OS was not reached in the pembrolizumab treatment group (HR = 0.641; 95% CI: 0.203-1.852; P=0.390; **Figure 3**). Subgroup analysis based on age, smoking status, tumor stage, PD-L1 expression level, and whether or not to combine chemotherapy revealed no significant differences in OS between patients in the sintilimab and pembrolizumab groups (**Figure 2B**).

### 3.4 Adverse reactions

The incidence of treatment-related AEs of any grade was 92.1% and 90.1% in the sintilimab and pembrolizumab groups, respectively, while the incidence of grade 3-4 AEs was 42.9% and 37.6%, respectively. The most common adverse reactions in the sintilimab group were alopecia (50.8%), constipation (36.5%), anemia (34.9%), and nausea (33.3%), while in the pembrolizumab group, the most common adverse reactions were alopecia (44.6%), constipation (41.6%), anemia (38.6%), and nausea (35.6%). The most common grade 3-4 AE in the sintilimab group was alopecia (19.1%). The most common grade 3-4 AEs in the pembrolizumab group were alopecia (11.9%) and reduced white blood cell count (11.9%). No patient had a grade 5 AE (**Table 3**).

**Table 3:** Comparison of adverse events between the two groups.

Comparison of adverse drug reactions between the two groups (n, %)				
Adverse event	Pembrolizumab+chemotherapy (N=101)		Sintilimab+chemotherapy (N=63)	
	All grades	Grade III-IV	All grades	Grade III-IV
Any terms	91(90.1%)	38(37.6%)	58(92.1%)	27(42.9%)
Alopecia	45(44.6%)	12(11.9%)	32(50.8%)	12(19.1%)
Anemia	39(38.6%)	8(7.9%)	22(34.9%)	7(11.1%)
White blood cell count decreased	32(31.7%)	12(11.9%)	20(31.7%)	8(12.7%)
Neutrophil count decreased	31(30.7%)	9(8.9%)	19(30.2%)	8(12.7%)
Platelet count decreased	20(19.8%)	5(5%)	12(19%)	3(4.8%)
Nausea	36(35.6%)	2(2%)	21(33.3%)	3(4.8%)
Vomiting	9(8.9%)	1(1%)	8(12.7%)	2(3.2%)
Decreased appetite	24(23.8%)	5(5%)	18(28.6%)	3(4.8%)
Constipation	42(41.6%)	3(3%)	23(36.5%)	3(4.8%)
Diarrhea	9(8.9%)	2(2%)	8(12.7%)	1(1.6%)
Transaminases increased	21(20.8%)	4(4%)	16(25.4%)	2(3.2%)
Fatigue	30(29.7%)	4(4%)	18(28.6%)	2(3.2%)
Peripheral neuropathy	23(22.8%)	5(5%)	15(23.8%)	3(4.8%)
Rash	12(11.9%)	2(2%)	6(9.5%)	2(3.2%)
Weight decreased	20(19.8%)	2(2%)	10(15.9%)	3(4.8%)

## 4 Discussions

Squamous lung cancer accounts for approximately 25%-30% of all lung cancers (9). Because of its unique clinical features, pathological manifestations and genetic mutation characteristics, squamous lung cancer is significantly different from lung adenocarcinoma in treatment and is often explored as a separate type in clinical studies. Patients with advanced squamous lung cancer are often unable to benefit from targeted therapy due to the lack of driver mutations (10, 11). Most patients with squamous lung cancer have a history of heavy smoking, resulting in complex genetic mutations and a high tumor mutational load (12). Complex mutations can cause neoantigen production, while a high tumor mutational load can drive effective antitumor immune responses and lead to a sustained clinical response to immunotherapy. These findings provide a rationale for lung squamous cancer patients to benefit from immunotherapy (13, 14).

In recent years, it has been shown that immunotherapy in combination with chemotherapy can lead to significant improvements in patient outcomes, possibly related to the immunological effects mediated by chemotherapeutic agents through direct and indirect stimulation of immune responses and increased tumor immunogenicity. Some clinical studies of immunotherapy combined with chemotherapy, such as KEYNOTE-407 (7), IMpower131 (15), and ORIENT-12 (16), compared the efficacy of immune-combination chemotherapy with chemotherapy alone. Treatment with a combination of paclitaxel/albumin paclitaxel + carboplatin and pembrolizumab significantly prolonged OS and PFS compared to chemotherapy alone (15.9 months vs. 11.3 months and 6.4 months vs. 4.8 months, respectively) (7). The IMpower131 study comparing atezolizumab combined with carboplatin and albumin-bound paclitaxel to chemotherapy alone in patients with stage IV squamous NSCLC revealed that the median OS in the ITT population was 14.2 months (95% CI: 12.3-16.8) vs. 13.5 months (95% CI: 12.2-15.1), HR=0.88 (95% CI: 0.73-1.05), p=0.158. However, in patients with high PD-L1 expression or in the TC3/IC3 subgroup, an OS advantage was seen with atezolizumab in combination with chemotherapy (23.4 months (95% CI: 17.8-NE) vs. 10.2 months (95% CI: 7.1-17.5), HR=0.48 (95% CI: 0.29-0.81) (15). In the ORIENT-12 study comparing the efficacy of the PD-1 inhibitor sintilimab in combination with gemcitabine and platinum versus chemotherapy alone, the median progression-free survival in the sintilimab in combination with gemcitabine and platinum group versus the chemotherapy alone group was 5.5 months and 4.9 months (HR=0.536, 95% CI: 0.422-0.681, p<0.00001), and the ORR in the sintilimab ORR in the combination chemotherapy group was 44.7% (16). Patients enrolled in a clinical trial (RCT) must meet the restrictions and criteria required by the trial, but as the availability of immunotherapeutic agents in oncology patients continues to increase, there are increasingly more patients in practice who do not meet the strict requirements of RCTs regarding treatment with these immunotherapeutic agents. The criteria in different clinical trials may not reflect the heterogeneity of real-world oncology populations. This study is based on real-world data and compares two agents with similar near-term efficacy, long-term survival benefit, and safety profile in the current Chinese clinical setting for treatment of patients with advanced squamous non-small cell lung cancer. To our knowledge, this study is the first real-world study to retrospectively compare treatment efficacy and safety of two PD-1 inhibitors in patients with advanced squamous lung cancer.

The values of ORR obtained in this study are similar to those obtained in previous clinical trials (4, 7, 16). In the pembrolizumab arm of this study, the ORR for squamous NSCLC was as high as 61.4%. The ORR for patients with squamous NSCLC in the sintilimab arm was 65.1%. In the pembrolizumab group, the DCR in squamous NSCLC patients was as high as 92.0%. The DCR in patients with squamous NSCLC in the sintilimab group was 92.0%. There was no statistically significant difference in ORR and DCR between the two drugs. Median OS data from the ORIENT-12 clinical trial are not yet available, and the median OS according to the KEYNOTE-407 Chinese population data was 30.1 months. The values of median OS in the two groups in our study were similar to the values arrived at in previous clinical trials. The median PFS in both groups in our study was longer than the PFS reported in the KEYNOTE-407 and ORIENT-12 clinical trials. In the pembrolizumab group, the median PFS for patients with squamous NSCLC was 16.5 months. In the sintilimab group, the median PFS for squamous NSCLC patients was 22.2 months, with no statistically significant difference in median PFS between the two groups. The following considerations may explain the phenomenon observed in the data of this study. First, the combined immune-drug chemotherapy regimen in the real world is different from the treatment regimen in clinical trials. Patients in the sintilimab group in this study received immune combination paclitaxel or albumin-bound paclitaxel and platinum regimens in approximately 92.1% of all patients, applied immune combination immune combination docetaxel and platinum in 3.2%, and applied immune combination gemcitabine and platinum in only 5%; this is different from the ORIENT-12 trial in which all patients used immune combination gemcitabine and platinum-based regimens and may have contributed to the differences in ORR and PFS in the sintilimab group in this study compared to the ORIENT-12 trial. It has been suggested that sintilimab combined with paclitaxel or albumin-bound paclitaxel chemotherapy may have similar clinical benefits compared to sintilimab combined with gemcitabine and platinum-based chemotherapy in patients with untreated advanced or metastatic squamous non-small cell lung cancer (17). In the pembrolizumab group in this study, immune therapy in combination with paclitaxel or albumin-bound paclitaxel and carboplatin regimens was received in a total of 17.8% of all patients, immune therapy in combination with paclitaxel or albumin-bound paclitaxel and cisplatin or loplatin in a total of 68.3%, immune therapy in combination with gemcitabine and platinum in 7%, and immune therapy in combination with docetaxel and platinum in tacitaxel and platinum in 7%, which is different from the KEYNOTE-407 trial in which all patients received immune therapy in combination with an albumin paclitaxel/paclitaxel + carboplatin regimen; this may have contributed to the differences in ORR and PFS values for patients in the pembrolizumab group in this study compared with the KEYNOTE-407 trial. Notably, there was no significant difference between ORR, PFS and OS in the two groups in this study. Second, the proportion of patients with high PD-L1 and positive PD-L1 expression in our study was much higher than that in the clinical trials (7, 16). Among patients who received PD-L1 expression assays prior to pembrolizumab treatment, 67.9% (36/53) were PD-L1 positive, exceeding the proportion reported in the KEYNOTE-407 clinical trial. Among patients who received PD-L1 expression assays prior to sintilimab treatment, 73.1% (19/26) were PD-L1 positive, exceeding the rate reported in the ORIENT-12 clinical trial study. PD-L1 has been found to be expressed at high levels in most NSCLC patients and appears to be a favorable prognostic factor for early-stage disease, and higher PD-L1 expression is associated with a survival benefit in NSCLC patients (18, 19); however, there remains a subset of patients with PD-L1 TPS < 1% who could benefit from immunotherapy alone, suggesting that PD-L1 is an imperfect predictive biomarker (20, 21).

It is worth noting that the binding targets and biological properties of the two drugs are not identical, which may account for the different ORR, PFS and OS values observed between the two groups of patients. Pembrolizumab is a humanized monoclonal antibody that binds to the programmed cell death protein 1 (PD-1) receptor on T cells and blocks its interaction with PD-L1 and PD-L2 ligands, which is a key immune checkpoint pathway. Pembrolizumab is composed of a human IgG4 kappa constant region and a murine anti-human PD-1 monoclonal antibody variable region (22). The predominant binding site for the combination of pembrolizumab and PD-1 is the C’D loop structure, which currently stands as the most efficacious PD-1/PD-L1 inhibitor in terms of affinity. The unique structure of pembrolizumab provides high specificity and affinity for PD-1, leading to potent immune checkpoint inhibition. Sintilimab, on the other hand, is a fully human monoclonal antibody that also targets the PD-1 receptor on T cells, but it has a different antibody structure from pembrolizumab. Sintilimab is composed of a human IgG4 kappa constant region and a fully human anti-human PD-1 monoclonal antibody variable region (23). The fully human structure of sintilimab is thought to potentially reduce the risk of immunogenicity and infusion reactions compared to pembrolizumab. The primary binding site for the combination of sintilimab and PD-1 is the FG loop structure. Both pembrolizumab and sintilimab are effective immunotherapies that target the PD-1 receptor. However, the difference in efficacy between the two drugs due to the difference in drug structure and biological properties is unclear yet.

Our study showed no significant difference between the two groups in terms of median PFS and OS. In terms of PFS and OS in patients with advanced squamous lung cancer, the results of these subgroup analyses in different strata of PD-L1 as well as in different age strata suggest that, clinically, sintilimab is not inferior to pembrolizumab. Both sintilimab and pembrolizumab may have common adverse drug reactions such as fatigue, rash, diarrhea and nausea. It is worth noting that the mechanism of action of both drugs is to activate the killing function of T cells by inhibiting PD-1/PD-L1 pathway to achieve the purpose of killing tumor. However, in the process of activating T cells, the difference of the binding targets and biological properties of the two drugs may interfere with differences in the incidence of adverse reactions between the two drugs. In our study, the spectrum of adverse reactions in the two groups in this study was generally similar to the spectrum of adverse reactions observed in previous clinical trials for both drugs, while the incidence of AEs of any grade and grade 3-4 AEs in this study was relatively consistent between the sintilimab and pembrolizumab groups; there were no significant differences, and the safety profiles were good.

There are some limitations to this study. First, this was a single-center retrospective study with a relatively small sample size. Therefore, information bias cannot be avoided, and the study results need to be further confirmed by retrospective or prospective studies that involve large samples. Second, due to the limited follow-up period, the median OS of patients in both groups in some subgroup analyses was not reached. We will further extend the follow-up period to refine the study data. Third, the PD-L1 expression level is now expected to be the first potential predictive biomarker to predict the outcome and prognosis of patients with advanced NSCLC (24, 25). Patients representing a particular subset of squamous NSCLC cases in this study did not undergo immunohistochemical PD-L1 testing for various reasons, and it is necessary to retrospectively analyze pathological samples from this population to expand the sample size for further analysis. In addition, treatment selection bias was inevitable in the two groups of patients in this study. In the real world, dosing and chemotherapy regimens cannot be administered in exactly the same way as used in clinical trials due to various factors. Although these factors somewhat attenuate the validity and reliability of the conclusions, the findings of this study are still highly relevant to the selection of clinical treatment regimens.

## 5 Conclusions

In summary, this study demonstrates that in real-world patients with advanced squamous lung cancer, first-line treatment with sintilimab in combination with chemotherapy is similar in near-term efficacy, long-term survival benefit and safety to combined treatment with pembrolizumab and chemotherapy.

## Data availability statement

The raw data supporting the conclusions of this article will be made available by the authors, without undue reservation.

## Ethics statement

Written informed consent was obtained from the individual(s) for the publication of any potentially identifiable images or data included in this article.

## Author contributions

WY: study design, experiments, and manuscript writing. WY, MG, TW, and LW: analysis and interpretation of data.WY, TL, YB, and YL: collection and interpretation of data. FZ, HT, JM, FJ, LW, and YH: interpretation of data and manuscript revision. All authors contributed to the article and approved the submitted version.
